# Prevalence of Potent Artificial Sweetener Neotame in Popular US-Marketed Disposable Electronic Cigarettes

**DOI:** 10.21203/rs.3.rs-6074654/v1

**Published:** 2025-02-27

**Authors:** Hanno C. Erythropel, Sairam V. Jabba, Remi A. Mellinghoff, Victor Garcia-Gallet, Peter Silinski, Julie B. Zimmerman, Sven E. Jordt

**Affiliations:** Department of Chemical and Environmental Engineering, Yale University, New Haven, Connecticut; Department of Anesthesiology, Duke University School of Medicine, Durham, North Carolina; Department of Chemical and Environmental Engineering, Yale University, New Haven, Connecticut; Rensselaer Polytechnic Institute, Troy, New York; Department of Chemistry, Duke University, Durham, North Carolina; Department of Chemical and Environmental Engineering, Yale University, New Haven, Connecticut; Department of Anesthesiology, Duke University School of Medicine, Durham, North Carolina

## Abstract

**Background:**

Sweeteners are used by tobacco product manufacturers to improve product palatability and appeal. Recently, the high-intensity artificial sweetener, neotame, was detected in nicotine analogue-containing and “zero nicotine” electronic cigarettes (e-cigarettes). The prevalence of neotame in popular US-marketed disposable e-cigarettes remains unknown.

**Methods:**

Popular disposable e-cigarette devices were chosen based on 2024 US market surveys and analyzed for neotame using Liquid Chromatography – Mass Spectrometry (LCMS). Nicotine, 6-methyl nicotine and cooling compounds were also quantified. Contents of FDA-approved e-cigarettes, nicotine analogue-containing, “zero-nicotine” disposables and a candy product were compared.

**Results:**

Neotame was detected in all 57 tested popular disposable e-cigarette devices, zero-nicotine, and nicotine analogue products, but not in 13 FDA-approved nor Juul products. 2 disposable products marketed in 2021 also contained neotame. The synthetic coolant WS-23 was detected in the large majority of tested products, with menthol and WS-3 less prevalent.

**Conclusions:**

The high prevalence of the artificial sweetener neotame in disposable e-cigarettes raises concerns about its effects on appeal and abuse potential of these products, especially in combination with cooling and sweet-associated flavors (fruit, candy) known to be preferred by youth. While more heat-stable than other artificial sweeteners, the degradation of neotame in e-cigarettes needs to be investigated and assessed for potential toxicity.

## Introduction

Sweeteners are used by tobacco product manufacturers to improve product palatability and appeal.^1^ We recently detected the high-intensity artificial sweetener, neotame, in electronic cigarettes (e-cigarettes) containing the nicotine analogue 6-methylnicotine.^2^ Neotame, an FDA-approved sweetener, is 7,000–13,000x sweeter than table sugar and differs from the better-known aspartame (200x) in one molecular feature (Figure). Besides a recent report on one “zero-nicotine“ brand,^3^ the prevalence of neotame addition to e-cigarette products is unknown. The aim of this study was to analyze current popular US-marketed disposable e-cigarettes, FDA-approved-, and nicotine-free products for this potent sweetener. Ancillary analysis included nicotine and nicotine analogues, menthol and synthetic cooling agents.

## Methods

Eleven popular disposable e-cigarette brands (27 devices) were chosen based on the 2024 National Youth Tobacco Survey and Empty Packs Survey USA-NY.^4,5^ Additionally, all currently US FDA-approved cartridge-based e-cigarettes (11), Juul (2), two nicotine analogue-containing products (20), and four “zero-nicotine” products (8) were tested (Table). In total, 68 products and one reference product (mint-candy) were purchased online in 2024 (Supplement-1). Further, two brands purchased in 2021 (4) were tested. Neotame was quantified both at Yale and Duke University laboratories using established methods.^2^ Briefly, an e-liquid aliquot was diluted using methanol or water and neotame content was quantified by liquid chromatography-mass spectroscopy. Samples tested at Yale were also analyzed by gas chromatography-flame ionization to quantify nicotine, 6-methylnicotine, menthol, and the synthetic coolants WS-3 and WS-23 using established methods.^2^

## Results

Neotame was detected in 57/72 tested vaping products, with an average neotame content ~ 4.6x higher than in a mint candy (1.75 vs. 0.38μg/mg). Neotame was detected in all products categorized as popular disposable, zero-nicotine, or nicotine analogue, while no FDA-approved cartridge nor Juul contained neotame. 2/4 samples from 2021 contained neotame (1/2 brands). Devices containing the nicotine analogue 6-methylnicotine contained the highest average neotame content albeit with large standard deviation (2.69 ± 3.91μg/mg). WS-23 was detected in the large majority of tested products (50/63), while menthol (28/63) and WS-3 (8/63) were less prevalent. No nicotine was detected in “zero-Nicotine” or nicotine analogue products.

## Discussion

This study demonstrates that the artificial sweetener neotame has become a ubiquitous constituent of popular disposable e-cigarettes sold in the US in 2024, including those containing no nicotine or nicotine analogues. Some vaping products contained neotame as early as 2021. However, FDA-approved devices (Vuse, NJOY) or awaiting approval (Juul), all introduced pre-2021, do not.

Sweetened tobacco products, and especially those with fruit and candy flavors are highly popular among youth and young adults.^5^ An experimental clinical study demonstrated that addition of the sweetener sucralose increased appeal, preference, and abuse potential of e-cigarettes.^6^ The presence of neotame, the most potent FDA-approved sweetener, may thus likely also increase the appeal and sensory attributes of flavored e-cigarettes in youth and young adults, an effect potentially synergistic with the presence of the synthetic coolant WS-23. The prevalence of neotame in almost all US-marketed, popular disposable e-cigarettes also raises toxicity concerns: While neotame was probably chosen for its heat stability relative to other sweeteners, It remains unclear to what extent it decomposes when heated and vaporized in e-cigarettes, and what the respiratory and systemic health effects of neotame and its decomposition products are. Study limitations include testing only a limited number of brands and flavors, although products were chosen based on popularity.

Current federal and state regulations do not limit the sweetener content of tobacco products, including e-cigarettes, enabling manufacturers to improve product appeal by increasing their sweetness. More information on neotame’s contribution to disposable cigarette appeal, abuse liability, and toxicity is needed to determine whether regulatory intervention is necessary.

## Figures and Tables

**Figure 1:**
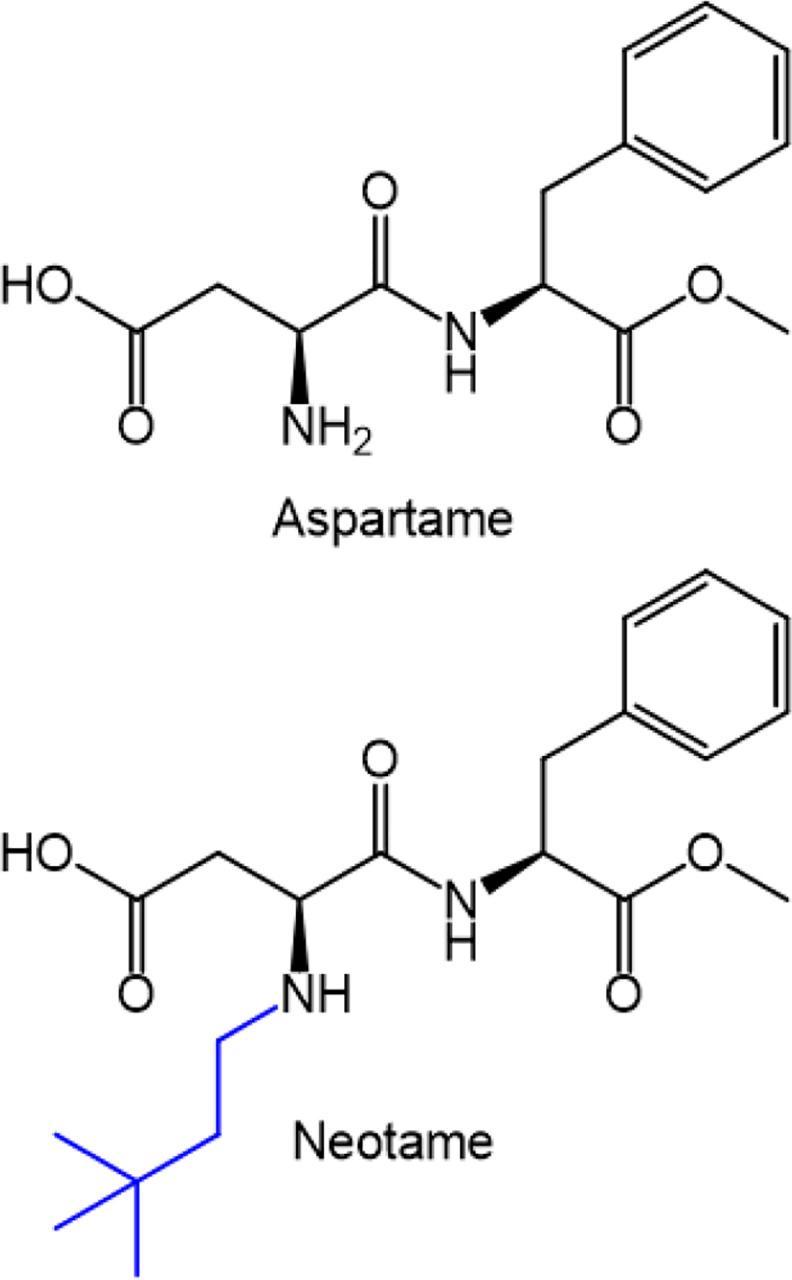
Chemical structures of aspartame and neotame, with the structural difference highlighted in blue.

**Table.** Tested vaping products broken down by product category (number of brands per category), number of products containing the artificial sweetener neotame, quantified mean (standard deviation), and measured range. Also included is information on the presence of nicotine, and the synthetic coolants WS-23 (Chemical Abstract Service, CAS No. 51115-67-4) and WS-3 (39711-79-0). All measurements n≥2. Detailed information available in Supplement 1. n.d. – not detected

Product category (# of brands)	Products containing neotame / total products	Neotame mean (SD) (μg/mg)	Neotame range in (μg/mg)	Nicotine and synthetic coolant presence (# of products not tested)
**Reference food product, mint candy (1)**	**1/1**	**0.38 (0.06)**		
**All tested vaping products**	**57/72**	**1.75 (2.49)**	**0 – 18.26**	
2024 Popular disposable devices (11)	27/27	1.12 (0.84)	0.12 – 2.80	Nicotine: 22/22 (5)
				Syn. coolants: 22/22 (5)
US FDA-approved cartridge devices (4) + Juul (1)	0/13	n.d.	n.d.	Nicotine: 13/13
				Syn. coolants: 1/13
Devices containing nicotine analogs (2)	20/20	2.69 (3.91)	0.20 – 18.3	Nicotine: 0/20
				Syn. coolants: 19/19 (1)
Devices marketed as “0 nicotine” (4)	8/8	1.79 (0.77)	0.48 – 2.95	Nicotine: 0/8
				Syn. coolants: 8/8
Re-tested 2021 devices (2)	2/4	0.10 (0.04)	0.07 – 0.13	(4)

converted from % to μg/mg); measured contents of nicotine (or 6-methylnicotine in []), neotame, menthol, WS-23, and WS-3 in μg/mg shown as mean (standard deviation) with n ≥ 2; and purchase information including vendor/supplier, data sorted by purchase date. Samples tested at Yale TCORS laboratory core except where noted with ^‡^. Abbreviations: 6MN: 6-methylnicotine; n.d.: not detected; “-” denotes “not measured”; SFN: “Substitute For Nicotine”; CT: Connecticut; NC: North Carolina; VT: Vermont; MFG: Manufacturing Information.

**Table:** 

Label Information			Measured contents						Purchase Information	
Product	Flavor	Label nicotine (% or mg)	Nicotine [or 6MN] (μg/mg)	Neotame *sweetener* (μg/mg)	Menthol *coolant* (μg/mg)	WS-23 *coolant* (μg/mg)	WS-3 *coolant* (μg/mg)	PG/GL ratio (%)*	Vendor	Purchase Date
***Mentos Clean Breath Intense Peppermint Candy***^f^ *(non-nicotine reference product)*			n.d.	*0.38 (0.06)*	-	-	-	-	Amazon.com	Dec. 2024
**AirBar Diamond** ^a^	Clear	50 mg	54.0 (2.8)	0.39 (0.02)	n.d.	31.0 (1.4)	n.d.	60/40	EBDesign.com	Sept. 2024
	Arctic Icy		48.9 (1.5)	0.31 (0.05)	2.6 (0.0)	20.7 (0.3)	n.d.	60/40		
	Summer Blast		40.7 (4.6)	1.85 (0.07)	n.d.	15.6 (1.4)	2.4 (0.2)	60/40		
	Watermelon Candy		49.2 (4.2)	2.38 (0.08)	n.d.	14.5 (1.1)	n.d.	55/45		
**AirBar Nex** ^a^	Mexican Mango	5% = 50 μg/mg	36.6 (2.9)	0.64 (0.03)	n.d.	15.8 (1.1)	n.d.	60/40		
	Clear		39.6 (3.0)	1.01 (0.01)	n.d.	30.6 (2.0)	n.d.	60/40		
	Unicorn		32.7 (2.9)	0.40 (0.04)	n.d.	16.8 (1.2)	n.d.	65/35		
**BC5000** ^a^^,^^d^	Beach Day	50 mg	42.3 (1.2)	0.52 (0.03)	n.d.	18.1 (0.5)	n.d.	40/60	Vapedeal.com	
	Clear		37.7 (0.2)	0.12 (0.01)	3.1 (0.3)	33.5 (0.4)	n.d.	60/40	EBDesign.com	
	Rinbo Cloudd	0 mg	n.d.	2.12 (0.14)	n.d.	10.1 (0.5)	n.d.	50/50		
**Breeze Smoke** ^a^	Watermelon Mint	5% = 50 μg/mg	58.9 (5.7)	1.27 (0.06)	n.d.	27.5 (2.4)	n.d.	55/45	Ejuicedb.com	
	Grape		65.0 (4.2)	2.80 (0.16)	n.d.	29.1 (1.4)	n.d.	50/50		
**ElfBar BC5000** ^a^	Rainbow Candy	50 mg	41.8 (4.0)	0.37 (0.01)	n.d.	18.8 (1.6)	n.d.	55/45	Vapedeal.com	
	Passionfruit Orange Guava		38.5 (2.6)	0.79 (0.02)	n.d.	12.4 (0.6)	n.d.	55/45	EBDesign.com	
**Geekbar Pulse** ^a^	Sour Mango Pineapple	5% = 50 μg/mg	-	2.44 (0.02)	-	-	-	-	Vapewh.com	
Label Information			Measured contents						Purchase Information	
**Lava** ^a^^,^^d^	Sex on the Beach	3% = 30 μg/mg	32.1 (0.4)	1.15 (0.06)	n.d.	24.0 (0.1)	2.2 (0.0)	60/40	Vapedeal.com	Sept. 2024
	Watermelon Bubble Gum	0%	n.d.	2.60 (0.18)	1.7 (0.0)	17.5 (0.4)	n.d.	65/35		
	Clear Ice	5% = 50 μg/mg	60.3 (0.7)	0.48 (0.04)	3.5 (0.1)	30.5 (0.4)	n.d.	55/45		
**Mr. Fog** ^a^	Banana Raspberry Ice^‡^	5% = 50 μg/mg	-	2.30 (0.12)	-	-	-	-	Vaperistas.com	
	Blue Raspberry Cherry Ice^‡^		-	2.18 (0.08)	-	-	-	-		
	Bubble Gum^‡^		-	2.10 (0.10)	-	-	-	-		
	Rainbow Blast^‡^		-	2.08 (0.02)	-	-	-	-		
**Myle** ^a^	Clear	50 mg	37.0 (0.9)	0.88 (0.08)	2.5 (0.0)	25.1 (0.4)	3.1 (0.0)	60/40	Vapedeal.com	
	Sweet Tobaooo		35.3 (0.5)	0.50 (0.03)	n.d.	8.4 (0.1)	n.d.	60/40		
	Melon Honeydew		35.0 (0.8)	0.44 (0.03)	n.d.	10 (2.6)	n.d.	60/40		
**Pacha Syn** ^a^	White Peach Ice	5% = 50 μg/mg	50.9 (1.9)	0.12 (0.02)	n.d.	8.8 (0.2)	n.d.	60/40	Ejuicedb.com	
	Strawberry Watermelon Ice		51.0 (1.0)	0.21 (0.02)	n.d.	4.3 (0.1)	n.d.	60/40		
**Strio x EBCreate** ^a^	Pacific Chill	50 mg	35.0 (3.6)	1.03 (0.07)	n.d.	16.1 (1.4)	n.d.	60/40		
	Fruit Trail		37.1 (0.8)	1.51 (0.09)	n.d.	14.4 (0.3)	n.d.	55/45		
**Vuse Alto** ^b^	Golden Tobacco	2.4% = 24 μg/mg	27.1 (0.9)	n.d.	n.d.	n.d.	n.d.	55/45		
	Golden Tobacco	1.8% = 18 μg/mg	20.0 (2.0)	n.d.	n.d.	n.d.	n.d.	55/45	Electrictobacconist.com	
	Menthol		20.5 (1.4)	n.d.	11.0 (0.1)	1.8 (0.1)	n.d.	55/45		
	Rich Tobacco	5% = 50 μg/mg	57.2 (3.8)	n.d.	n.d.	n.d.	n.d.	55/45		
**Vuse Vibe** ^b^	Menthol	3% = 30 μg/mg	35.2 (3.1)	n.d.	12.9 (0.3)	n.d.	n.d.	30/70		
	Original		35.4 (1.3)	n.d.	n.d.	n.d.	n.d.	30/70		
**Vuse Solo** ^b^	Menthol	4.8% = 48 μg/mg	52.2 (4.6)	n.d.	7.6 (0.0)	n.d.	n.d.	30/70		
	Original		52.7 (4.0)	n.d.	n.d.	n.d.	n.d.	30/70		
Label Information			Measured contents						Purchase Information	
**NJOY Daily** ^b^	Menthol	4.5% = 45 μg/mg	48.5 (3.8)	n.d.	10.8 (0.1)	n.d.	n.d.	60/40	Electrictobacconist.com	Sept. 2024
	Extra Menthol	6% = 60 μg/mg	63.1 (1.2)	n.d.	11.1 (0.0)	n.d.	n.d.	60/40		
	Rich Tobacco	4.5% = 45 μg/mg	44.7 (1.6)	n.d.	n.d.	n.d.	n.d.	60/40		
	Extra Rich Tobacco	6% = 60 μg/mg	59.7 (3.3)	n.d.	n.d.	n.d.	n.d.	60/40		
**Sensa**^d^ [1]	Berry Fusion	0%	n.d. [n.d.]	2.95 (0.25)	3.8 (0.0)	12.2 (0.2)	n.d.	55/45	In-store, Durham, NC	July 2024
	Blueberry Frost			1.62 (0.20)	8.2 (0.4)	36.3 (0.1)	7.9 (0.1)	60/40		
	Mint Frost			1.18 (0.26)	0.91 (0.0)	35.0 (1.6)	7.8 (0.3)	60/40		
	Passionfruit Frost			1.79 (0.10)	11.5 (0.1)	18.9 (0.8)	7.9 (0.2)	60/40		
	Watermelon Frost			2.15 (0.34)	4.0 (0.0)	19.9 (0.3)	8.0 (0.1)	65/35		
**Kumi Six** ^c^	Banana Ice	5% “SFN” 50 μg/mg	[3.9 (0.1)]	3.66 (0.17)	0.4 (0.0)	18.8 (0.5)	n.d.	55/45	Ejuicedb.com	June/Nov. 2024
	Blue Razz		[4.4 (0.1)]	2.83 (0.23)	0.5 (0.0)	18.0 (0.3)	n.d.	60/40		
	Blue Razz Ice		[4.4 (0.1)]	3.13 (0.13)	0.5 (0.0)	13.4 (0.2)	n.d.	60/40		
	Cool Mint		[4.3 (0.1)]	1.83 (0.09)	7.3 (0.1)	24.8 (0.5)	n.d.	60/40		
	Mystery Ice (MFG: 122023)		[4.9 (0.1)]	18.3 (1.02)	0.4 (0.0)	18.2 (0.3)	n.d.	65/35		
	Mystery Ice (MFG: 32024)^‡^		-	3.8 (0.07)	-	-	-	-		
	Peach Ice		[4.4 (0.1)]	2.97 (0.10)	0.4 (0.0)	17.7 (0.2)	n.d.	60/40		
	Sourpunch Bluestraws		[4.1 (0.0)]	3.17 (0.08)	0.4 (0.0)	16.9 (0.2)	n.d.	60/40		
	Strawberry Fanta		[4.3 (0.0)]	2.96 (0.02)	0.4 (0.0)	19.0 (0.2)	n.d.	55/45		
	Strawberry Watermelon		[4.4 (0.0)]	3.34 (0.07)	0.4 (0.0)	14.5 (0.2)	n.d.	60/40		
	Watermelon Ice		[4.4 (0.1)]	3.06 (0.23)	0.5 (0.0)	18.9 (0.3)	n.d.	60/40		
**Loon Maxx** ^d^	Blue 0% Lightning	0%	n.d.	1.18 (0.11)	1.2 (0.0)	10.0 (1.0)	4.4 (0.4)	60/40	Supplied by VT Dept. of Health	June 2024

Label Information			Measured contents						Purchase Information	
**Juul** ^b^	Menthol	5% = 50 μg/mg	57.2 (3.6)	n.d.	7.6 (0.0)	n.d.	n.d.	40/60	Local CT gas station	Feb. 2024
	Virginia Tobacco		63.1 (5.7)	n.d.	n.d.	n.d.	n.d.	40/60		
**SpreeBar**^c^ [2]	Blood Orange Peach	5% “Metatine” = 50 μg/mg	[6.27 (0.49)]	0.56 (0.03)	n.d.	4.44 (0.04)	n.d.	60/40	Westsidevapor.com	Jan. 2024
	Blue Razz Ice		[5.82 (0.46)]	0.86 (0.12)	n.d.	16.39 (0.11)	n.d.	60/40		
	Creamy Melon		[6.09 (0.12)]	0.21 (0.01)	n.d.	5.74 (0.15)	n.d.	60/40		
	Pineapple Coconut		[5.97 (0.26)]	0.60 (0.03)	n.d.	4.47 (0.03)	n.d.	60/40		
	Rainbow Fruit		[6.17 (0.09)]	0.65 (0.02)	n.d.	4.34 (0.07)	n.d.	60/40		
	Strawberry Apple Melon		[6.06 (0.47)]	0.20 (0.02)	n.d.	14.01 (0.18)	n.d.	60/40		
	Strawberry Mango		[5.94 (0.35)]	0.25 (0.01)	n.d.	9.81 (0.07)	n.d.	60/40		
	Sweet Spearmint		[6.11 (.026)]	0.56 (0.01)	3.5 (0.1)	16.18 (0.17)	n.d.	55/45		
	Watermelon Grape		[6.20 (0.67)]	0.51 (0.11)	n.d.	4.81 (0.05)	n.d.	60/40		
**Geek bar** ^e^	Icy Cola^‡^	5% = 50 μg/mg	-	0.07 (0.00)	-	-	-	-	Bigdvapor.com	Aug. 2021
	Grape Soda^‡^		-	0.13 (0.01)	-	-	-	-		
**Puffbar** ^e^	Blueberry Ice^‡^		-	n.d.	-	-	-	-	Eliquidstop.com	
	Watermelon^‡^		-	n.d.	-	-	-	-		

* rounded to the nearest 5%;

‡ Tested at Duke University;

a-f refer to the product category that devices were sorted into as presented in the manuscript table: a - 2024 Popular disposable devices, b - US FDA-approved cartridge devices + Juul, c – devices containing nicotine analogs, d – devices marketed as “0 nicotine“, e – re-tested 2021 devices, f – reference food product;

References:

[1] Robichaud MO, Talbot EM, Ganz O, et al. Marketing for Sensa: a 'zero nicotine vapor producť from a major tobacco company. Tobacco Control. 2025;doi: https://doi.org/10.1136/tc-2024-059074

[2] Erythropel HC, Jabba SV, Silinski P et al. Variability in Constituents of E-Cigarette Products Containing Nicotine Analogues. Jama. Sep 3 2024;332(9):753–755. doi: https://doi.org/10.1001/jama.2024.12408
